# Mixed Dentition Analysis Using Moyers' and Tanaka-Johnston's Methods in the Indian Population: A Systematic Review

**DOI:** 10.7759/cureus.81208

**Published:** 2025-03-25

**Authors:** Arya Binu, Kavitha Ramar

**Affiliations:** 1 Department of Pedodontics and Preventive Dentistry, SRM Kattankulathur Dental College and Hospital, Chennai, IND

**Keywords:** indian population, mixed dentition, moyers analysis, orthodontic treatment planning, tanaka-johnston analysis

## Abstract

Mixed dentition analysis has been vital for the early identification and management of malocclusion in pediatric dentistry. This systematic review evaluates the effectiveness of Moyers' and Tanaka-Johnston's methods for predicting the mesiodistal width of unerupted canines and premolars in the Indian population. A computerized search of articles published on PubMed and Scopus was conducted. The search resulted in a sample composed of 328 articles, out of which 14 were selected. The results show that the predictability of these analyses among the study populations varied, with a 20-30% acceptance rate for Moyers' analysis compared to Tanaka-Johnston's method. The majority of the papers analyzed indicated that the accuracy of Tanaka-Johnston's method did not meet expectations when compared to Moyers' analysis.

## Introduction and background

Pedodontists have a wonderful opportunity to encounter patients who are still growing. One of the main issues during dentofacial development is malocclusion. In the mixed dentition phase, an appropriate space evaluation can be used to address this issue early. To decide whether the orthodontic treatment plan will include serial extraction, space maintenance, space regaining, or just periodic patient monitoring, mixed dentition analysis is a crucial factor [1]. A precise estimation of the mesiodistal width of the unerupted permanent teeth is necessary to determine the tooth size-arch length discrepancy in the mixed dentition [2].

Three categories exist for space analysis in mixed dentition: regression equations, radiography, or a combination of the two. Using a regression equation, the Moyers (1958) and Tanaka-Johnston (1974) methods determine the mesiodistal width of unerupted teeth; the Nance (1947), Bull (1959), and Huckaba (1964) methods measure the unerupted teeth using radiographs; and the Hixon and Oldfather (1958) and Staley and Karber (1980) methods combine the first two techniques. Tanaka-Johnston's and Moyers' methods are the most frequently utilized among all the analyses [2-4].

Mandibular permanent incisors are the first to erupt, have less variation in shape and size, are easy to measure, and have a high correlation with other groups of teeth. Hence, most methods use them to predict the sum of the mesiodistal dimensions of the canines and premolars [3]. In Moyers' analysis, a likelihood table was established using the sum of the mesiodistal diameters of the mandibular incisors. To prevent potential crowding, the author recommends using the table at the 75% probability level. The probabilities vary from 5% to 95%. Some benefits of this approach include minimal systematic error, ease of use, safety for both novices and experts, speed, no need for radiography, and the ability to perform the procedure directly in the mouth. On the other hand, the Tanaka-Johnston analysis employs regression equations [4,5].

However, the applicability of these analyses across different ethnic populations, including Indians, has been questioned due to potential variations in dental morphology. Despite several investigators proposing different space analysis methods for various ethnic groups within the Indian population, a systematic review assessing the accuracy of Moyers' and Tanaka-Johnston's analyses specifically for the Indian population has not been conducted [6-8].

This systematic review sought to address this gap by consolidating current evidence to assess how accurately Moyers' and Tanaka-Johnston's analyses predict the mesiodistal width of unerupted canines and premolars in the Indian population.

## Review

Methods

The search strategy was carried out in accordance with the Cochrane Handbook for Systematic Reviews of Interventions [9]. A computerized search was conducted by searching articles published on PubMed (http://www.ncbi.nlm.nih.gov/pubmed/) and Scopus (https://www.scopus.com/sources.uri?zone=TopNavBar&origin=searchbasic). This systematic review adhered to the Preferred Reporting Items for Systematic Reviews and Meta-Analyses (PRISMA) checklist. The study protocol was registered with the International Prospective Register of Systematic Reviews (PROSPERO) under the protocol number CRD42024562119.

The search terms incorporated both controlled vocabulary, Medical Subject Headings (MeSH), and uncontrolled vocabulary (text words and their synonyms). Boolean operators (AND, OR, NOT) were used to combine search terms, and the strategy was adapted for each database. The keywords used and the corresponding number of articles found are shown in Table 1, with the searches conducted without restricting the publication period.

**Table 1:** Keyword search strategy.

Keywords	PubMed	Scopus	Other
Mixed dentition	761	1641	719
Orthodontic treatment planning			
Moyers analysis			
Tanaka Johnston analysis			
Indian population			

Inclusion criteria included studies involving the Indian pediatric population, studies with Moyers' mixed dentition analysis compared with Tanaka-Johnston's analysis, studies including randomized controlled trials (RCTs), and clinical trials involving mixed and permanent dentition. Exclusion criteria included studies with analyses other than Moyers' and Tanaka-Johnston's methods, studies not conducted in the Indian population, systematic reviews and meta-analyses, and studies on primary dentition.

Before the start of data extraction and other methods, the keywords were checked for appropriateness, and the search category was tested for the reliability and validity of the articles. During the initial screening phase, two reviewers (AB and KR) independently assessed the relevance of titles and abstracts based on predefined inclusion and exclusion criteria. In the full-text review phase, the same two reviewers independently examined each article to confirm its adherence to the inclusion criteria. They also extracted comprehensive details regarding the study methodologies, interventions, comparisons, and outcomes. This rigorous review resulted in 14 studies that were deemed eligible for qualitative synthesis.

Data extraction was performed using a standardized form, capturing key details such as study authors, year, country, study design, the objective of the study, and the outcome measures. This structured approach ensured that only relevant studies were thoroughly reviewed and synthesized to provide insights into the use of mixed dentition analyses in the Indian population.

Results

A total of 3,126 articles constituted the sample. A total of 1,185 papers were chosen for screening after being examined for duplication and other reasons; 328 were selected for the review process. A total of 182 articles were assessed for eligibility, and finally, 14 articles were deemed eligible to be included in the study. Table 2 displays the number of selected publications and the systematic review's methodological requirements.

**Table 2:** Studies included and excluded after reviewers’ analyses.

Keywords - PubMed database	Studies included	Studies excluded	Total
432	118	314	9
Keywords - Scopus database	Studies included	Studies​​​​​​​ excluded	Total
753	210	543	5

A total of 970 articles were eliminated (duplicates published in other databases), and 650 articles did not meet the eligibility criteria. Table 3 lists the reasons for exclusion, as well as the total number of publications that were eliminated.

**Table 3:** Reasons for articles exclusion.

Reason for exclusion	PubMed	Scopus	Total
Reason 1 (not in the Indian population)	30	21	51
Reason 2 (did not use both Moyers' and Tanaka-Johnston's analyses)	54	20	74
Reason 3 (insufficient data reporting)	11	27	38
Reason 4 (systematic review and meta-analysis)	1	4	5
Total number of studies excluded			168

**Figure 1:**
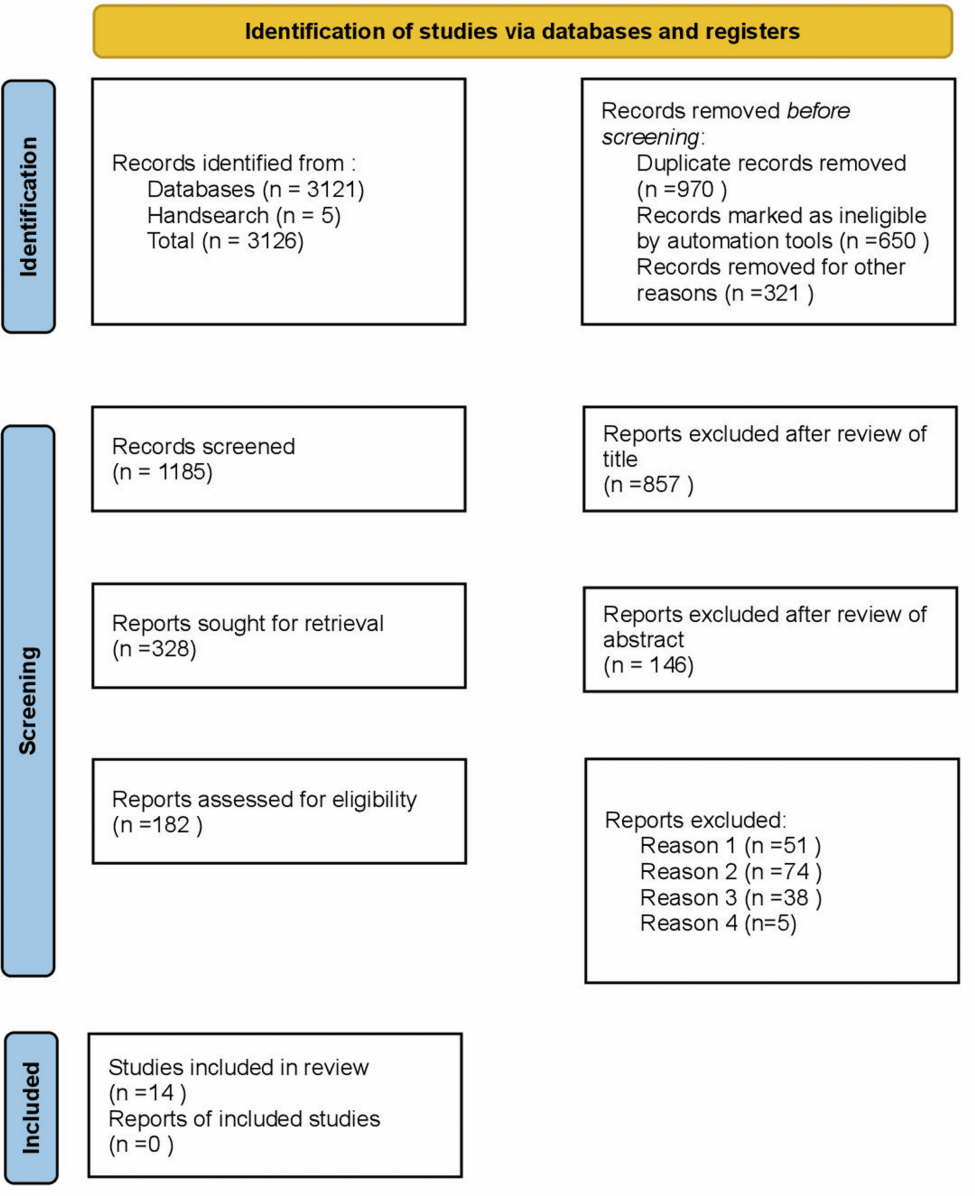
PRISMA flow diagram detailing the study identification and selection process. PRISMA: Preferred Reporting Items for Systematic Reviews and Meta-analyses.

A total of 182 articles were assessed for eligibility. Finally, 14 articles were included for data extraction (Figure 1).

Table 4 summarizes the articles included in the study.

**Table 4:** Summarized data of the 14 articles. max.: maxillary; mand.: mandibular; UCPM: upper canine premolar; LCPM: lower canine premolar; mdw: mesiodistal width; M: molar; I: incisor; C: canine; P: premolar; r: correlation coefficient; T&J: Tanaka-Johnston.

Articles	Study design	Demography	Outcome measure	Results correlation coefficient	Conclusion
1. Shobha et al. (2016) [10]	Cross-sectional study	50 males, 50 females; age range: 13-15 years	Mesiodistal dimensions of mandibular incisors, maxillary and mandibular permanent canines, and first and second premolars were measured from dental casts of the samples.	Females: max. arch = 0.49, mand. arch = 0.38 ; males: both arches = 0.33; both male and female combined in max. arch = 0.56, both male and female combined in mandibular arch = 0.523	Overestimation in both arches in Tanaka-Johnston analysis. Overestimation in both arches in Moyers (75th percentile). In males, the predicted values were accurate, and in females, underestimation in the maxillary arch. Males had close predictions; females exhibited overestimation in the mandibular arch in Moyers (50th percentile).
2. Sholapurmath et al. (2012) [11]	Cross-sectional study	Age range: 13-16 years; gender distribution: 70 males, 70 females	Mesiodistal dimensions of mandibular permanent incisors, maxillary and mandibular permanent canines, and first and second premolars were measured from dental casts of the samples.	UCPM: males = 0.5617, females = 0.3657, total = 0.4356; LCPM: males = 0.6868, females = 0.4297, total = 0.5499	Underestimation of both Moyers' and Tanaka-Johnston's methods. In males, the significantly greater size of the maxillary canine-premolar segment.
3. Dasgupta et al. (2012) [12]	Cross-sectional study	Age range: 11 to 14 years; gender distribution: 36 females, 34 males	Mesiodistal dimensions of all erupted permanent incisors, canines, and premolars were measured from the dental casts.	Mand. arch = 0.611; max. arch = 0.591	Overestimation of both Tanaka-Johnston's and Moyers' methods.
4. Ravinthar et al. (2020) [13]	Cross-sectional study	Age range: 11 to 15 years; gender distribution: 500 males, 500 females	The sum of mesiodistal measurements of mandibular incisors and the combined width of maxillary canines and premolars measured on both quadrants from the dental casts.	Not mentioned	Overestimation in both genders of both Tanaka-Johnston's and Moyers' methods.
5. Kommineni et al. (2014) [14]	Not mentioned	Age range: not mentioned; gender distribution: 127 females, 343 males	All teeth from the left second premolar to the right second premolar of each set of dental casts were measured.	Not mentioned	Overestimation by Tanaka-Johnston. In Moyers, slightly more accurate prediction.
6. Sonahita et al. (2012) [15]	Cross-sectional study	Age range: 13 to 21 years; gender distribution: 100 males, 100 females	Measurements of mesiodistal diameter of all permanent mandibular and maxillary canines and premolars and mandibular incisors and first molars were measured from the dental casts.	Males - UCPM: 0.66 lower incisors, 0.80 lower incisors + lower molars; LCPM: 0.61 lower incisors, 0.73 lower incisors + lower molars. Females - UCPM: 0.67 lower incisors, 0.75 lower incisors + lower molars; LCPM: 0.57 lower incisors, 0.70 lower incisors + lower molars	Underestimation - Moyers method. Overestimation - Tanaka-Johnston method.
7. Ramesh et al. (2014) [16]	Cross-sectional study	Age range: 14 to 20 years; gender distribution: 30 males, 30 females	The teeth measured included mandibular permanent incisors, maxillary and mandibular permanent canines, and first and second premolars from dental casts.	Females: max. arch = 0.52, mand. arch = 0.60; males: max. arch = 0.60, mand. arch = 0.46; both male and female combined in max. arch = 0.58; both male and female combined in mandibular arch = 0.59	Tanaka-Johnston – Overestimated for both genders. Moyers at 75% overestimated values more in males than in females.
8. Grover et al. (2017) [17]	Cross-sectional study	Age range: 11-15 years; gender distribution: 100 boys, 100 girls	The sum of mesiodistal measurements of mandibular incisors, combined width of mandibular canines and premolars, and combined width of maxillary canines and premolars measured on both quadrants from dental casts.	Not mentioned	Males - Moyers overestimated, Tanaka-Johnston underestimated. Females - Moyers underestimated, Tanaka-Johnston overestimated.
9. Manjula et al. (2013) [18]	Cross-sectional study	Age range: 13 to 16 years; gender distribution: 100 males, 100 females	Mesiodistal dimensions of mandibular permanent incisors, maxillary and mandibular permanent canines, and the first and second premolars were measured from dental casts.	Females - 0.40 = lower, 0.25 = upper; males - 0.59 = lower, 0.46 = upper; both sexes combined: 0.50 = lower, 0.36 = upper arch	Overestimation by Tanaka-Johnston methods in both males and females. Moyer’s prediction tables can be used at all probability levels for male and female subjects.
10. Doda et al. (2021) [19]	Cross-sectional study	Age range: 11-15 years; gender distribution: 100 males, 100 females	Mesiodistal width of permanent mandibular incisors, right maxillary and mandibular canines, and right maxillary and mandibular first and second premolars measured from dental casts.	Males - 0.528 mand. canine and premolars, 0.597 max. canine and premolars; females - 0.517 mand. canine and premolars, 0.520 max. canine and premolars	Males - Moyers overestimated, Tanaka-Johnston underestimated. Females - Moyers underestimated.
11. Goyal et al. (2014) [20]	Not mentioned	Age range: 14-22 years; gender distribution: first phase - 80 males, 80 females; second phase - 50 males, 50 females	Mesiodistal width of permanent mandibular incisors, canine, first premolar, and second premolar in both maxillary and mandibular arches taken from study models.	Males: 0.52 sum of mdw of mand. I (max. arch), 0.72 sum of mdw of mand. I and mand. 1st M (max. arch), 0.58 sum of mdw of mand. I (mand. arch), 0.76 sum of mdw of mand. I & mand. 1st M (mand. arch); females: 0.61 sum of mdw of mand. I (max. arch), 0.87 sum of mdw of mand. I and mand. 1st M (max. arch), 0.74 sum of mdw of mand. I (mand. arch), 0.89 sum of mdw of mand. I & mand. 1st M (mand. arch)	Tanaka-Johnston overestimated.
12. Kamatham et al. (2017) [21]	Cross-sectional study	Age range: 11-15 years; gender distribution: 100 males, 101 females	Mdw of mandibular incisors, maxillary and mandibular canines, and premolars measured from dental casts.	Actual mesiodistal widths of maxillary canines and premolars - males: r = 0.63 Tanaka-Johnston, r = 0.61 (Moyers 35%), r = 0.57 (Moyers 75%); females: r = 0.65 Tanaka-Johnston, r = 0.66 (Moyers 35%), r = 0.64 (Moyers 75%); actual mesiodistal widths of mandibular C and Ps - males: r = 0.67 Tanaka-Johnston, r = 0.64 (Moyers 35%), r = 0.62 (Moyers 75%); females: r = 0.64 Tanaka-Johnston, r = 0.64 (Moyers 35%), r = 0.62 (Moyers 75%)	Overestimation - Tanaka-Johnston's method. Overestimation with Moyers at 75% level and underestimation at 35% level.
13. Bhatnagar et al. (2019) [22]	Cross-sectional study	Age range: 11 to 14 years; gender distribution: 60 males, 60 females	The greatest mesiodistal dimension of each tooth was measured between its contact point from dental casts.	Not mentioned	Overestimation by Tanaka-Johnston's method and Moyers at 75th percentile.
14. Suma et al. (2020) [23]	Cross-sectional study	Age range: 16-23 years; gender distribution: 200 males, 200 females	Mesiodistal width measurement of mandibular permanent incisors, maxillary and mandibular permanent canines, first and second premolars.	Males: 0.46 mandibular arch, 0.60 maxillary arch; females: 0.60 mandibular arch, 0.52 maxillary arch; males + females (combined): 0.59 (mandibular arch) (T&J 0.65), 0.58 (maxillary arch) (T&J 0.62)	Overestimation by both methods. The percentage of overestimation was higher for Tanaka-Johnston than for Moyers (75%).

Shobha et al. (2016) and Sholapurmath et al. (2012) had similar findings regarding the gender-based approach, revealing that the male population exhibited variations due to tooth size, which influenced the model analysis [10,11]. The remaining studies showed that, in most cases, Tanaka-Johnston overestimated results compared to Moyers' analysis, and this tendency was more prevalent in the male population. Moyers' analysis is known to be reliable and accurate and had fewer implications of underestimating the analysis results compared to Tanaka-Johnston's method in females. The overall summary indicates that Moyers' analysis has a better predictive capacity than Tanaka-Johnston's, based on the compilations of the review.

Risk of bias (ROB) assessment

Cross-sectional analytical studies (observational and descriptive in nature with retrospective data) were included, with Moyers' analysis as the intervention component and Tanaka-Johnston's analysis as the comparator. The population considered was the Indian pediatric population.

This risk of bias (ROB) assessment was conducted to evaluate the accuracy between the two methods in estimating mixed dentition. The outcome measured was the association in the prediction of the mesiodistal width of unerupted canines and premolars in both arches (gender-wise and combined) while using the two mixed dentition analysis methods (Moyers' vs. Tanaka-Johnston's methods).

A Revised Risk of Bias Assessment Tool for Non-randomized Studies of Interventions (ROBANS 2) was used to evaluate the risk of bias in the included cross-sectional studies by one reviewer. It consists of eight domains: comparability of target groups, target group selection, confounders, measurement of intervention/exposure, blinding of assessors, outcome assessment, incomplete outcome data, and selective outcome reporting.

A decision was made by the reviewer to follow the criteria in each domain, pointing to a desired study design. The ROB was evaluated in each study based on criteria for each domain as "low," "high," or "unclear" risk of bias, specifically for cross-sectional studies in domains 1 and 2. Criteria for non-randomized studies were considered in domains 3, 5, and 7. Criteria for domains 4, 6, and 8 were considered in their original state. The overall risk of bias using ROBANS 2 was yielded by considering the worst risk of bias in any of the domains or any critical bias.

**Figure 2:**
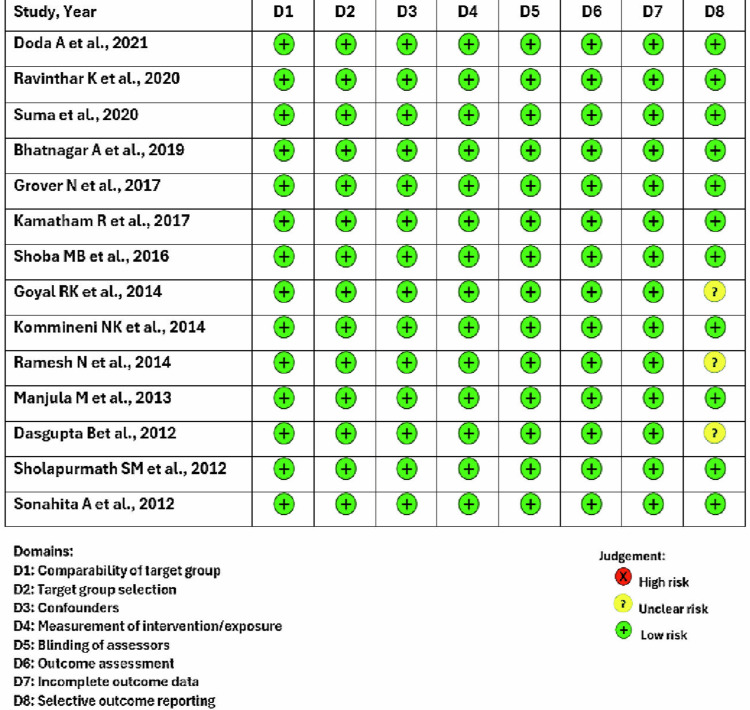
Traffic light plot using ROBANS 2 tool for risk assessment of the included cross-sectional studies. ROBANS 2: Revised Risk of Bias Assessment Tool for Non-randomized Studies of Interventions. References: Doda et al. [19], Ravinthar et al. [13], Suma et al. [23], Bhatnagar et al. [22], Grover et al. [17], Kamatham et al. [21], Shobha et al. [10], Goyal et al. [20], Kommineni et al. [14], Ramesh et al. [16], Manjula et al. [18], Dasgupta et al. [12], Sholapurmath et al. [11], Sonahita et al. [15].

**Figure 3:**
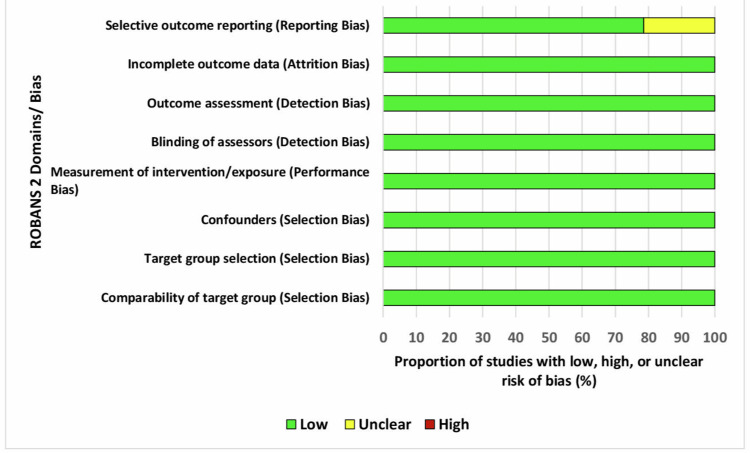
Summary bar plot using ROBANS 2 tool for risk assessment of the included cross-sectional studies. ROBANS 2: Revised Risk of Bias Assessment Tool for Non-randomized Studies of Interventions.

The domain-wise ROB judgments in each study were presented using a traffic light plot (Figure 2), and the proportion of studies within each type of bias was presented as a summary bar plot (Figure 3). The plots were graphically prepared using Microsoft Excel (Microsoft Corporation, Redmond, WA).

Among the included studies (n = 14), all showed a "low risk" in the first seven domains, and three studies had an "unclear risk" in domain 8 (Figure 2). The critical domains of bias for this systematic review were selection bias, performance bias, and detection bias, based on the hypothesis, study design, and methodology of the included studies. The summary bar plot (Figure 3) depicts that all the studies showed 100% "low risk" in the occurrence of selection bias (due to inappropriate comparison target groups, inappropriate intervention or exposure group selection, and inappropriate confounder confirmation and consideration); performance bias (due to inappropriate intervention or exposure measurement); detection bias (due to inappropriate blinding of assessors and outcome assessment methods); and attrition bias (due to inappropriate handling of incomplete data). About 21.43% of studies were assessed as having "unclear risk" in reporting bias (due to selective outcome reporting).

The overall risk of bias for the critical domains suggests an overall low risk, thereby favoring the strength of the hypothesis, study design, and methodology to predict the mesiodistal width of unerupted canines and premolars in both arches during the mixed dentition stage using Moyers' and Tanaka-Johnston's methods.

Discussion

To support our therapeutic approach, a systematic review is a literature evaluation focused on a specific research issue. The goal of the inclusion and exclusion criteria for this review was to select only the most pertinent publications on the topic at hand. When diagnosing and planning orthodontic treatment for individuals with mixed dentition, mixed dentition analysis is an essential step. The mesiodistal width of unerupted canines and premolars can be overestimated or underestimated, which can affect treatment planning. Therefore, using the wrong approach may compromise the overall treatment plan [10,11].

In this review, the evidence showed that the accuracy of Moyers' and Tanaka-Johnston's methods varied among the critically appraised articles, providing insights into the prediction accuracy of both methods. According to Table 4, Shobha et al. (2016) indicated that both methods overestimated results across both arches, particularly in terms of gender variations between males and females [10]. Males showed better accuracy with Moyers' analysis compared to Tanaka-Johnston's; however, in females, both methods demonstrated overestimation [12-16].

According to Sholapurmath et al. (2012), both methods predicted an overall underestimation, but greater variation was observed in the male population regarding the size of the maxillary canine and premolar segments [11]. Meanwhile, Dasgupta et al. (2012) showed that both methods yielded significantly overestimated results in both genders using cast models [12]. This finding coincided with the results from Ravinthar et al. (2020), who also indicated significant overestimation for both genders using both Moyers' and Tanaka-Johnston's methods [13]. Additionally, other studies conducted in 2012 and 2014 revealed varying measurement segments, with one study showing overestimation with Tanaka-Johnston's method and underestimation with Moyers' method during model analysis [16-18].

Ramesh et al. (2014) indicated that in the male population, both methods overestimated results by up to 75% [16]. In contrast, females exhibited less overestimation, but when males and females were combined, a similarity was observed in both arches with no significant variations. Similarly, Grover et al. (2017) and Doda et al. (2021) presented alternate results, stating that in the male population, Moyers' analysis was overestimated while Tanaka-Johnston's was underestimated. In females, the opposite trend was observed. Meanwhile, Manjula et al. (2013) stated that Moyers' method was more accurate than Tanaka-Johnston's, with similar probabilities for both genders [17,19,20].

Studies conducted with high accuracy by Kamatham et al. (2017) demonstrated that the actual width of both arches showed overestimation with Tanaka-Johnston's method and underestimation with Moyers at the 75% level, while at the 35% level, both methods represented underestimation [21]. These findings were echoed by Bhatnagar et al. (2019) [22]. According to Suma et al. (2020), both Moyers' and Tanaka-Johnston's methods exhibited overestimation in prediction analysis for both genders, with a greater variation percentage noted for Tanaka-Johnston's compared to Moyers' analysis [23].

The risk of bias was plotted using the ROBANS 2 tool, indicating a low risk in a positive manner to strengthen the review hypothesis. It demonstrated that there is variation in the accuracy of prediction analysis when using both methods, with Moyers being more favorable for estimating prediction accuracy in most studies.

Currently, Moyers' method (1963) is one of the most commonly used approaches, although its accuracy has recently come under scrutiny, primarily because its probability table was created based on a population with a predominance of Northern European ancestry. Recent studies have demonstrated that the prediction tables developed by Moyers are neither accurate nor appropriate for use with populations of diverse ethnic backgrounds, as the values at the 75% level do not match the actual values [21-23].

When the anticipated values at 75% are less than the true values, a significant clinical issue arises, as there may not be enough room to adequately align the teeth. Conversely, when the values overestimate the actual values, there will be greater space to accommodate the posterior teeth, which does not present a true clinical issue.

The review was limited to observational studies. Although the discussed studies have shown reliable results, further experimental designs are required for a more accurate approach in clinical practice.

## Conclusions

This systematic review assesses the accuracy of Moyers' and Tanaka-Johnston's methods for predicting unerupted tooth width during mixed dentition analysis. Both methods often overestimate, but when critically appraised, Moyers' analysis shows better prediction accuracy compared to Tanaka-Johnston's method. Overestimation is manageable clinically, but underestimation poses risks to alignment. The review highlights the need for population-specific research and customized prediction tables for better orthodontic planning.
